# Burden, social support, and coping strategies in family caregivers of individuals receiving home mechanical ventilation: a cross-sectional study

**DOI:** 10.1186/s12912-024-02024-6

**Published:** 2024-05-22

**Authors:** Lucyna Płaszewska-Żywko, Izabela Fajfer-Gryz, Jakub Cichoń, Maria Kózka

**Affiliations:** 1 Department of Clinical Nursing, Faculty of Health Sciences, Jagiellonian University – Medical College, ul. Kopernika 25, Krakow, 31-501 Poland; 2 Clinical Department of Angiology, University Hospital in Krakow, Krakow, Poland

**Keywords:** Home mechanical ventilation, Coping, Burden, Social support

## Abstract

**Background:**

Home mechanical ventilation (HMV) is a treatment method for patients with chronic respiratory failure. HMV is a challenge for both patients and their caregivers. Some studies have shown a higher risk of depression, job loss, or lifestyle disturbance in family members caring for HMV patients. The purpose of the study was to measure caregiver burden, perceived social support, and coping strategies.

**Methods:**

In the cross-sectional study, 58 caregivers (65.52% female) of HMV patients treated at five healthcare institutions were surveyed. The questionnaires including patient/caregiver demographic data, the type and duration of MV, the Caregiver Burden Scale (CBS), the Social Support Scale (SSS), and the Brief-COPE questionnaire were applied. For statistical analysis, the Mann‒Whitney U test, the Kruskal‒Wallis H test, Dunn’s test, and Spearman correlations were used.

**Results:**

In the CBS, a high level of burden was observed mainly in the isolation and disappointment subscales. The female caregivers achieved a higher score for general strain than did the male caregivers (*p* = 0.023). Differences in the distribution of the isolation (*p* = 0.028) and disappointment (*p* = 0.03) variables between the financial situation groups were observed. The older the patient, the lower the burden in the domains of isolation (*p* = 0.015) and disappointment (*p* = 0.005) was elicited. Invasive MV was associated with greater burdens of general strain (*p* = 0.005), isolation (*p* = 0.001), and disappointment (*p* = 0.001). A medium total SSS score was shown (74.5 ± 7.56). Caregivers used various coping strategies. The most common were planning, acceptance, and active coping. Self-blame and denial were positively related to several CBS subscales, whereas acceptance of difficult situations and positive reframing were related to lower scores.

**Conclusions:**

Caregivers most frequently experienced a medium level of burden. Female caregivers were characterized by higher level of burden. Invasive ventilation increased the burden. Respondents who assessed their financial situations as good, had lower burden in the selected subscales. Using some positive coping strategies may reduce the level of burden. Interventions to ensure that caregivers overcome these burdens should be undertaken.

## Introduction

Long-term mechanical ventilation (LTMV) is a method for treating chronic respiratory failure. LTMV is administered invasively or non-invasively. Invasive mechanical ventilation is based on the use of a respirator and tracheostomy tube, whereas non-invasive ventilation includes oronasal (preferred), full-face, or nasal masks, on a daily basis. According to definition, LTMV must be carried out daily for a period of at least three months. Treatment takes place mainly in homes or long-term facilities [1, 2].

Home mechanical ventilation (HMV) is one of the LTMV methods and its goal is to increase life expectancy and improve quality of life [2]. Patients can be assigned to HMV only when causal treatment is completed [3]. Common indications for HMV treatment include neuromuscular diseases (e.g., Duchenne muscular dystrophy, Becker muscular dystrophy, and myasthenia gravis), obstructive lung diseases (e.g., Chronic Obstructive Pulmonary Disease), and obesity-related respiratory failure [1]. Increasing numbers of patients who require prolonged mechanical ventilation at home, despite the benefits of this form of treatment, are challenging for both patients and their caregivers [4].

Polish institutions that hold a contract with the only public payer National Health Fund (*pol. Narodowy Fundusz Zdrowia*) provides patients with medical equipment and medical care, consisting of physician, nurse and physiotherapist visits, with the frequency tailored to the patient’s needs. However, patient family commitment is essential [3]. This can create a variety of difficulties for family members and other informal caregivers.

In the literature there is a limited number of studies that comprehensively assess the burden of caregivers of mechanically ventilated patients at home. Studies on burden of caregivers of patients who have left the intensive care unit (ICU) after acute respiratory failure are closest to this topic. Previous studies have shown that informal caregivers of ICU survivors have a high level of burden and greater risk of depression, post-traumatic stress disorder (PTSD), job loss, or lifestyle disturbance [5].

In Poland, research on the burden on caregivers has been undertaken only selectively, focusing on fatigue, coping strategies, or difficulties in caring for patients ventilated mechanically at home [6, 7].

Considering the importance of this issue and the limited number of studies on this subject we conducted research to fill the gap. The primary aim of our study was to measure caregiver burden, perceived social support, and coping strategies in caregivers of HMV patients and the relationship between these factors. The main research question was “Is there a relationship between burden, social support and coping strategies among family caregivers of HMV patients?”.

## Methods

### Study design

This cross-sectional study was conducted using the STROBE reporting guidelines in five healthcare institutions that provide services to HMV patients. An anonymous online survey and paper forms were sent to the caregivers. The approval of the institutions involved was obtained for this study.

### Population and sample

Family caregivers of HMV patients were recruited from various parts of Poland. The inclusion criteria included caring for adult HMV patients and providing informed consent. Caring for a person under 18 years of age, failure to be a primary caregiver, and lack of informed consent were exclusion criteria. Of the 300 questionnaires sent out through institutions providing care for mechanically ventilated patients at home and having a contract with the National Health Fund, 72 questionnaires were returned (response rate 24%). Fourteen of them were excluded from the study due to incomplete data or because respondents met the exclusion criteria. Finally, 58 fully completed questionnaires were included in the study.

### Questionnaires

The structured questionnaire included demographic data from the HMV patients and their caregivers, and the type and duration of mechanical ventilation were recorded. The Caregiver Burden Scale, the Social Support Scale, and the Brief-COPE were used.

The Caregiver Burden Scale (CBS) by Elmståhl et al. [8] contains 22 items in five subscales: general strain (8 items), isolation (3 items), disappointment (5 items), emotional involvement (3 items), and environment (3 items). In this study, the Polish version by Jaracz et al. [9] was used. The scale allows to evaluate the subjective burden experienced by caregivers of chronically disabled people, and the data are expressed as the mean value for the individual subscales. The severity of the burden can be expressed by the following criteria: low for a mean score between 1 and 1.99 points, average for 2.00-2.99 points, and high for 3.00-4.00 points. The reliability and validity of the original tool were acceptable, with Cronbach’s α between 0.70 and 0.87 (except for Environment, where Cronbach’s α = 0.53). The reliability and validity of the Polish adaptation were satisfactory, with a Cronbach’s α = 0.89 [8, 9].

The Social Support Scale (SSS) developed by Kmiecik-Baran [10] is designed to measure the perception of social support. The scale contains 24 items in 4 subscales: informational support, instrumental support, valuing support, and emotional support. The items are all scored on a five-point Likert scale. The total score ranges between 24 and 120 points. The higher the score is, the lower the social support received. The reliability of the SSS scale was satisfactory, with a Cronbach’s α ranging from 0.51 to 0.87. The original version of this questionnaire was used in the present study [10].

The Brief-COPE developed by Carver [11] evaluates stress coping strategies. In this study, the Polish adaptation by Juczyński and Ogińska-Bulik [12] was used. The questionnaire contains 28 items, scores from 0 to 3, and helps to reveal which of 14 stress coping strategies are implemented by the participants. The higher the score is, the more often the specific coping strategy is used by the respondent. The Cronbach’s α of the original scale ranged from 0.48 to 0.94, and the Polish adaptation had a Cronbach’s α ranging from 0.62 to 0.89. The split-half reliability of the Polish version was 0.86 [11, 12].

### Data analysis

Data analysis was performed using R statistical software version 3.3.2, and normal distribution testing was performed using the Shapiro‒Wilk test. To compare variables, the Mann‒Whitney U test and the Kruskal‒Wallis H test were applied. Post hoc analysis was performed using Dunn’s test. Nonparametric Spearman correlations were applied to analyze relationships between scales. The level of statistical significance was set at *p* < 0.05.

### Ethical considerations

The study was carried out in accordance with the Declaration of Helsinki. The study was approved by the Bioethics Committee of the Jagiellonian University, Krakow, Poland (No KBET/25/B/2014). Participants provided informed consent after being informed about the aim and the course of the study and were assured of their anonymity and the right to withdraw.

## Results

### Participants characteristics

The study group consisted of 58 caregivers with a mean age of 53.81 (SD ± 13.73) years (95% Confidence Interval: 50.28 to 57.34), and most of them were females (65.52%). In contrast, the HMV patients were mainly males (51.72%), the mean age of the patients was 56.47 years (SD ± 14.62; 95% Confidence Interval: 52.71 to 60.23), and the patients were mostly (63.79%) invasively ventilated. The average duration of mechanical ventilation (MV) was 3.54 years (SD ± 2.63; 95% Confidence Interval: 2.86 to 4.22). The characteristics of the participants and patients are detailed in Table 1.

**Table 1:** Characteristics of the participants and caregiver burden (*n* = 58)

		General strain		Isolation		Disappointment		Emotional involvement		Environment	
	n (%)	x̅ (SD)	*p - value*	x̅ (SD)	*p - value*	x̅ (SD)	*p - value*	x̅ (SD)	*p - value*	x̅ (SD)	*p - value*
**Caregivers**											
Gender											
female	38 (65.52%)	2.60 (0.70)	0.023^*^	2.79 (0.85)	0.081^*^	2.83 (0.90)	0.028^*^	1.89 (0.83)	0.435^*^	1.94 (0.68)	0.498^*^
male	20 (34.48%)	2.63 (0.83)		2.36 (0.78)		2.30 (0.77)		1.68 (0.61)		2.13 (0.86)	
Marital status											
married	41 (70.69%)	2.60 (0.70)	0.925^*^	2.57 (0.80)	0.576^*^	2.58 (0.86)	0.324^*^	1.82 (0.74)	0.903^*^	2.00 (0.74)	0.979^*^
single	17 (29.31%)	2.63 (0.83)		2.80 (0.94)		2.82 (0.95)		1.82 (0.84)		2.02 (0.79)	
Place of residence											
town	38 (65.52%)	2.52 (0.78)	0.187^*^	2.63 (0.86)	0.869^*^	2.60 (0.88)	0.549^*^	1.79 (0.82)	0.388^*^	2.00 (0.67)	0.836^*^
rural	20 (34.48%)	2.76 (0.63)		2.66 (0.83)		2.74 (0.92)		1.88 (0.67)		2.01 (0.90)	
Education											
primary	8 (13.79%)	2.61 (0.82)	0.961^**^	2.54 (0.69)	0.553^**^	2.35 (0.83)	0.365^**^	1.71 (0.76)	0.263^**^	2.37 (1.17)	0.677^**^
vocational	10 (17.24%)	2.68 (0.67)		2.70 (0.78)		2.92 (0.88)		2.10 (0.67)		1.80 (0.67)	
secondary	18 (31.03%)	2.54 (0.68)		2.48 (0.79)		2.47 (0.90)		1.90 (0.82)		1.96 (0.73)	
higher	22 (37.93%)	2.62 (0.82)		2.79 (0.97)		2.78 (0.89)		1.66 (0.76)		2.00 (0.60)	
Livelihood											
work	20 (34.48%)	2.73 (0.73)	0.278^**^	2.75 (0.81)	0.461^**^	2.70 (0.91)	0.305^**^	1.76 (0.78)	0.696^**^	2.23 (0.73)	0.158^**^
disability benefits^a^	12 (20.69%)	2.65 (0.74)		2.52 (1.00)		2.65 (0.80)		2.00 (0.78)		2.11 (0.83)	
pension	15 (25.86%)	2.31 (0.73)		2.42 (0.76)		2.33 (0.90)		1.78 (0.90)		1.86 (0.77)	
others	11 (18.97%)	2.74 (0.74)		2.88 (0.85)		2.98 (0.89)		1.78 (0.58)		1.66 (0.54)	
Subjective financial situation											
good	23 (39.66%)	2.42 (0.79)	0.237^**^	2.32 (0.95)	**0.028** ^**^	2.31 (0.94)	0.030^**^	1.78 (0.76)	0.191^**^	1.93 (0.67)	0.534^**^
average	28 (48.28%)	2.67 (0.66)		2.74 (0.64)		2.77 (0.78)		1.72 (0.70)		2.13 (0.82)	
bad or very bad	7 (12.06%)	2.94 (0.77)		3.33 (0.77)		3.26 (0.75)		2.33 (0.93)		1.76 (0.68)	
**Patients**											
Gender											
female	28 (48.28%)	2.51 (0.77)	0.255^*^	2.75 (0.89)	0.466^*^	2.75 (0.89)	0.606^*^	1.69 (0.75)	0.146^*^	2.02 (0.71)	0.688^*^
male	30 (51.72%)	2.69 (0.70)		2.54 (0.80)		2.54 (0.80)		1.94 (0.77)		1.99 (0.79)	
Type of MV											
invasive	37 (63.79%)	2.82 (0.72)	0.005^*^	2.14 (0.86)	0.001^*^	2.15 (0.83)	0.001^*^	1.81 (0.77)	0.928^*^	1.81 (0.77)	0.452^*^
noninvasive	21 (36.21%)	2.23 (0.61)		2.93 (0.69)		2.93 (0.8)		1.83 (0.77)		1.83 (0.77)	

*Abbreviations: n *sample size,  *x̅* arithmetic mean, *SD* standard deviation

^*^Mann‒Whitney U test

^**^Kruskal‒Wallis test

^a^including: Carer’s allowance

### Burden

In the subscale general strain, 37.93% of participants ranked their burden as average and 36.21% as high. As shown in Table 2, a high level of burden was chosen most often in the isolation and disappointment subscale, and emotional involvement was chosen least often.

**Table 2:** Level of caregiver burden (*n* = 58)

Domain	Level of burden	*n* (%)
General strain	low	15 (25.86%)
	average	22 (37.93%)
	high	21 (36.21%)
Isolation	low	10 (17.24%)
	average	22 (37.93%)
	high	26 (44.83%)
Disappointment	low	13 (22.41%)
	average	20 (34.48%)
	high	25 (43.10%)
Emotional involvement	low	36 (62.07%)
	average	14 (24.14%)
	high	8 (13.79%)
Environment	low	26 (44.83%)
	average	23 (39.66%)
	high	9 (15.52%)

*Abbreviation*: *n *sample size

The female caregivers achieved a significantly (*p* = 0.023) greater score in the domain general strain than did the male caregivers (2.77 vs. 2.29), which indicates that females perceived themselves as more burdened.

Considering financial status, statistical significance was not revealed in the general strain subscale (*p* = 0.237); however, differences in the distribution of the isolation (*p* = 0.028) and disappointment (*p* = 0.03) variables between the financial situation groups were observed. Post hoc analysis showed that respondents who rated their financial status as good had a lower burden on this subscale than did those who rated it as bad or very bad.

Patient age had a statistically significant impact on the scores in the domains of isolation (*p* = 0.015) and disappointment (*p* = 0.005), as it was shown that the older the patient was, the lower the burden. Invasive MV was also shown to be associated with greater burdens on the subscales of general strain (*p* = 0.005), isolation (*p* = 0.001), and disappointment (*p* = 0.001) than was non-invasive mechanical ventilation (NIMV).

No statistically significant differences were detected between general strain and age (*p* = 0.173), marital status (*p* = 0.925), place of residence (*p* = 0.187), education (*p* = 0.961), professional activity (*p* = 0.641), livelihood (*p* = 0.278), patient gender (*p* = 0.255) or period of MV in years (*p* = 0.198) (Table 1).

### Social support

According to the Social Support Scale (SSS), the average total score was 74.5 (SD = 7.56), and caregivers gave the highest rating to informational support and the lowest to valuing support. The SSS scores were not related to those obtained for any of the CBS subscales (Table 3).

**Table 3:** Social support and burden (*n* = 58)

Social Support Scale								General strain from CBS	
Domain	x̅	SD	Me	Min	Max	Q1	Q3	R	*p* - value
Informational	17.81	2.24	18	12	25	17	19	0.021	0.876
Instrumental	18.83	3.26	18	11	28	17	22	-0.085	0.528
Valuing	19.16	2.75	19	13	29	18	20.75	0.163	0.222
Emotional	18.71	2.34	18	14	27	18	19	0.106	0.430
Total score	74.50	7.56	74.5	63	104	69	79	0.069	0.609

*Abbreviations: n *sample size, *x̅ *arithmetic mean, *SD *standard deviation, *Me *median, *Min *minimal value, *Max *maximum value, *Q1 *lower quartile, *Q3 *upper quartile, *R *value of the Spearman’s rank correlation coefficient

### Coping

In response to stress, caregivers used various strategies, and among all planning, acceptance, and active coping were the most common. In contrast, the least commonly used were substance use, humor, and behavioral disengagement. The study showed that the more frequently the respondents used a self-blaming strategy, the greater their disappointment (*p* = 0.032) and emotional involvement (*p* = 0.002) were. It was also revealed that the more frequently the denial strategy was presented, the greater the emotional involvement (*p* = 0.001), and the more frequently the acceptance of difficult situations was presented, the lower the general strain (*p* = 0.007), isolation (*p* = 0.001), disappointment (*p* = 0.031), and emotional involvement (*p* = 0.041). Furthermore, it was revealed that the more frequent the use of positive reframing, the lower the isolation was (*p* = 0.027). The other relationships were not statistically significant (Table 4).

**Table 4:** Coping strategies and burden (*n* = 58)

Brief-COPE				Caregiver Burden Scale									
				General strain		Isolation		Disappointment		Emotional involvement		Environment	
Strategy	x̅ (SD)	Me (Q1-Q3)	Min – Max	R	*p* - value	R	*p* - value	R	*p* - value	R	*p* - value	R	*p* - value
Active coping	2.16 (0.64)	2.00 (2.00-2.50)	0.5-3	-0.028	0.835	0.116	0.385	0.012	0.930	-0.056	0.674	-0.245	0.064
Planning	2.22 (0.65)	2.00 (2.00-2.88)	0.5-3	-0.112	0.403	-0.095	0.478	-0.100	0.455	-0.216	0.103	-0.096	0.473
Positive reframing	1.86 (0.74)	2.00 (1.50–2.50)	0–3	-0.218	0.101	**-0.290**	**0.027**	-0.216	0.104	-0.151	0.259	-0.135	0.311
Acceptance	2.18 (0.65)	2.00 (2.00-2.50)	0–3	**-0.349**	**0.007**	**-0.416**	**0.001**	**-0.283**	**0.031**	**-0.269**	**0.041**	-0.074	0.582
Humor	0.72 (0.62)	0.50 (0.12-1.00)	0-2.5	0.039	0.772	0.163	0.223	0.096	0.475	0.136	0.309	0.001	0.991
Religion	1.46 (1.01)	1.75 (0.62–2.38)	0–3	0.054	0.689	0.012	0.932	0.098	0.464	0.001	0.993	0.003	0.985
Use of emotional support	2.03 (0.84)	2.00 (1.50–2.50)	0–3	-0.176	0.187	-0.240	0.070	-0.206	0.122	-0.055	0.683	-0.128	0.340
Use of instrumental support	1.98 (0.75)	2.00 (1.50–2.50)	0.5-3	-0.064	0.634	-0.191	0.151	-0.101	0.449	-0.056	0.678	-0.186	0.162
Self-distraction	1.52 (0.81)	1.50 (1.00–2.00)	0–3	0.035	0.792	0.139	0.299	0.152	0.255	0.233	0.079	0.035	0.793
Denial	1.08 (0.82)	1.00 (0.50–1.50)	0–3	0.253	0.056	0.072	0.593	0.136	0.307	**0.408**	**0.001**	0.238	0.072
Venting	1.32 (0.78)	1.50 (1.00-1.88)	0–3	0.143	0.283	0.237	0.073	0.158	0.237	0.131	0.327	-0.059	0.662
Substance use	0.41 (0.67)	0.00 (0.00–1.00)	0-2.5	0.029	0.829	0.026	0.846	-0.013	0.925	0.084	0.529	0.125	0.349
Behavioral disengagement	0.82 (0.67)	1.00 (0.50-1.00)	0-2.5	0.163	0.221	0.150	0.261	0.192	0.149	0.223	0.092	0.005	0.968
Self-blame	1.03 (0.81)	1.00 (0.50–1.50)	0–3	0.105	0.434	0.159	0.232	**0.283**	**0.032**	**0.391**	**0.002**	-0.087	0.518

*Abbreviations: n *sample size,  *x̅ *arithmetic mean, *SD *standard deviation, *Me *median, *Q1 *lower quartile, *Q3 *upper quartile, *Min *minimal value, *Max *maximum value, *R *value of the Spearman’s rank correlation coefficient

## Discussion

The specificity of caring for MV patients may have a negative impact on the functioning of family caregivers, causing lifestyle disruptions, depression, increased overload, daily life fatigue and risk of losing one’s job and worsening physical health [5, 7, 13]. We focused on examining the burden among family caregivers of individuals mechanically ventilated at home as well as factors related to both care recipients (e.g. type of MV) and the caregivers (e.g. sociodemographic and economic factors), as studies show that they may influence the burden [14]. We also investigated coping strategies and social support in caregivers and the relationships between these three variables in order to obtain a more comprehensive assessment of factors that affect caregiver burden.

In our study, the caregivers were primarily women, and the patients were mainly men. The same relationship has been shown in other studies [5, 15–18].

### Burden

Caregiver burden is a multidimensional challenge influenced by many factors [19]. Our study showed that most caregivers had an average level of burden. In comparison, caregivers from Canada experienced a high average level of burden, reporting that the most difficult time they experienced was the first couple months after their loved ones returned home [20]. Similar results were achieved in South Korea, where studies showed that family caregivers had a high level of burden that could impact their quality of life [17, 21].

A large burden was shown in German caregivers of invasively ventilated patients with Amyotrophic Lateral Sclerosis (ALS) [22], and in Italy, where more than half of HMV patients met at least two of the five criteria established by the researchers as conditions that are major causes of caregiver burden [23]. One-third of Spanish caregivers of HMV patients were found to be overburned or at risk of strain [16], and the study conducted by Van Pelt et al. [5] suggested that caregiver burden was high and did not depend on pre-ICU patient functional status. Chiò et al. [24] proved that in the group of caregivers of patients with ALS (both non-invasive ventilated and tracheostomized), the level of burden increases as the patient’s disability deteriorates.

The opposite results were obtained by Tsara et al. [15] among caregivers of NIMV patients in Greece. Most of the participants rated their burden as light on all subscales (employment issues, household management, financial issues, social relations), similar to the findings of Siciliano et al. [25] in the group of caregivers of patients with ALS (with 35% NIMV patients), and most respondents showed a low level of burden.

A study conducted in Taiwan and Israel revealed that caregivers of HMV patients experience a greater level of burden than caregivers of patients staying in chronic respiratory care facilities [18, 26].

Our study showed that females presented a higher burden level in CBS significantly more frequently than males, and a similar relationship was revealed in several studies [18, 24, 27]. A review by Adelman et al. [28] also indicated that female gender is a risk factor for chronic patient caregivers. Male caregivers of MV patients in South Korea presented a greater burden than females did; however, these relationships were not statistically significant [17].

Our research also revealed that the use of invasive ventilation had a significantly greater burden on caregivers than did the use of NIMV. Similar conclusions were reached by Van Kesteren et al. [29], Kaub-Wittemer et al. [22], and Yotani et al. [30] in a group of caregivers of children older than 15 years. However, different results were obtained by Kim et al. [21].

### Social support

In our study, the participants valued information support the most. However, no significant correlations were found between perceived social support and burden. Some research shows that social support is crucial for the functioning of caregivers, as in the study by Boettcher et al. [31], where it was shown that social support was a significant predictor of mental health for mothers of pediatric patients requiring long-term mechanical ventilation. Likewise, in the meta-analysis by Del-Pino-Casado et al. [32], in caregivers of adults and older adults burden was negatively related to perceived and received social support. Furthermore, in the clinical review by Adelman et al. [28], burden was a risk factor for social isolation. According to Liu et al. [18], healthcare personnel should provide social support to reduce burdens and ease stress among caregivers. Liang et al. [33] indicated that people who receive greater social support are more willing to choose HMV. Additionally, a study by Nadig et al. [34] showed that family members of ICU survivors developed more frequent adaptive coping behaviors and presented lower rates of depression, anxiety, and posttraumatic stress if provided with better social and economic support.

### Coping

The families of HMV patients are characterized by a high level of stress, which also has a significant impact on deterioration of quality of life [35]. In our research, we focused on determining how caregivers respond to stress. Most often, the participants used positive strategies such as planning, acceptance, and active coping. Similar results were obtained by Pérez-Cruz et al. [36], where caregivers of elderly dependent relatives most often applied coping strategies, such as acceptance, active coping and the use of emotional support. Among caregivers of patients in the vegetative state and in the minimally conscious state, positive coping strategies (active coping, use of instrumental support, planning, and acceptance) also prevailed. Maladaptive coping strategies such as denial or self-blame were associated with anxiety and depression, whereas acceptance was associated with their absence [37]. Our study revealed that the more frequent the acceptance of difficult situations was, the lower the general burden was.

Previous Polish studies have indicated that the greater the degree of daily life fatigue is, the more often caregivers of HMV patients experience strategies of avoidance and manifested helplessness [7]. Other research has shown that caregivers seek support from their families and friends and resort to religion in difficult situations [6].

Studies among families of patients receiving home NIV indicated that respondents most often used coping strategies related to the reorientation of goals, resignation, and passivity [16]. Other research also conducted among German caregivers of ALS patients treated with home NIV revealed acceptance and denial as the most common coping strategies [38], whereas caregivers of patients with ALS in Italy most often adopted task-oriented coping strategies. Furthermore, it was stated that there was a significant association between emotion-oriented coping strategies and high levels of burden and anxiety symptoms [25]. Tramonti et al. [39] showed that among hospitalized patients with ALS, the coping strategies of avoidance and venting were positively related to the physical burden of caregivers. As shown in research by Petrinec [40], among family decision makers of patients placed in long-term acute care hospitals, a problem-focused coping strategy was most often applied, and the least commonly used was an avoidant coping strategy. Additionally, an avoidant coping strategy was moderately associated with signs and symptoms of anxiety and PTSD [40].

## Conclusions

In our study group, most of the caregivers were female, while the majority of the patients were male. Female caregivers were characterized by the higher level of burden in CBS. Invasive ventilation in patients increased the burden. It was shown that adaptive coping strategies, such as acceptance and positive reframing, may reduce the level of burden. There was no statistically significant relationship between caregiver burden and perceived social support.

The results of our study revealed the extent to which caregivers of HMV patients are burdened and how specific factors affect it. In view of the negative impact of this burden on caregivers’ lives, macro- and microlevel evidence-based psychological, educational, financial and organizational interventions should be implemented. Further research is needed to comprehensively examine difficulties and challenges of caregivers and evaluate the effectiveness of various strategies (e.g. respite care) to reduce the burden of carers and thus improve their functioning.

### Limitations

The limitations of the study include the relatively low number of participants and the fact that some of the questionnaires (less than 14%) were completed online. The representativeness of the sample could be questionable, as only 24% of caregivers responded to the questionnaires. This may lead to bias and makes it impossible to extrapolate results. Another limitation is that the CBS used in our research was validated in Poland among caregivers of stroke survivors, not in mechanically ventilated patients, and invariance of the scale across gender was not examined. Although the results confirm the data of some other authors, they do not allow for unambiguous conclusions to be drawn regarding the greater burden on women. It should be noted that the research instruments used have been self-reported and characterized by the examination of subjective perception. Inaccuracies or misunderstandings could affect the accuracy of the data collected.

## Data Availability

The datasets used and analyzed during the current study are available from the corresponding author on reasonable request.

## References

[1] Park S, Suh ES (2020). Home mechanical ventilation: back to basics. Acute Crit Care.

[2] Markussen H, Lehmann S, Nilsen RM, Natvig GK (2018). Factors associated with change in health-related quality of life among individuals treated with long-term mechanical ventilation, a 6-year follow-up study. J Adv Nurs.

[3] Butna K, Pyszora A, Adamczyk A, Krajnik M (2021). Practical aspects of nursing care provided to patients diagnosed with amyotrophic lateral sclerosis receiving home mechanical ventilation. Palliat Med Pract.

[4] King AC (2012). Long-term home mechanical ventilation in the United States. Respir Care.

[5] Van Pelt DC, Milbrandt EB, Qin L, Weissfeld LA, Rotondi AJ, Schulz R, Chelluri L, Angus DC, Pinsky MR (2007). Informal caregiver burden among survivors of prolonged mechanical ventilation. Am J Respir Crit Care Med.

[6] Stodulska M, Biłogan L (2016). Wybrane aspekty jakości życia chorych wentylowanych mechanicznie w warunkach domowych oraz ich opiekunów. Nurs Anaesthesiol Intensive Care/Pielęgniarstwo w Anestezjologii i Intensywnej Opiece.

[7] Szatkowska K, Szkulmowski Z (2018). Daily life fatigue and coping strategies in family caregivers of home mechanically-ventilated individuals. Palliat Med Pract.

[8] Elmståhl S, Malmberg B, Annerstedt L (1996). Caregiver’s burden of patients 3 years after stroke assessed by a novel caregiver burden scale. Arch Phys Med Rehabil.

[9] Jaracz K, Grabowska-Fudala B, Kozubski W (2012). Caregiver burden after stroke: towards a structural model. Neurol Neurochir Pol.

[10] Kmiecik-Baran K (1995). Skala Wsparcia Społecznego. Teoria i wartości psychometryczne. Przegląd Psychologiczny.

[11] Carver CS (1997). You want to measure coping but your protocol’s too long: consider the brief COPE. Int J Behav Med.

[12] Juczyński Z, Ogińska-Bulik N (2012). Narzędzia Pomiaru Stresu I Radzenia Sobie Ze Stresem.

[13] Douglas SL, Daly BJ (2003). Caregivers of long-term ventilator patients: physical and psychological outcomes. Chest.

[14] Loo YX, Yan S, Low LL (2022). Caregiver burden and its prevalence, measurement scales, predictive factors and impact: a review with an Asian perspective. Singap Med J.

[15] Tsara V, Serasli E, Voutsas V, Lazarides V, Christaki P (2006). Burden and coping strategies in families of patients under noninvasive home mechanical ventilation. Respiration.

[16] Fernández-Alvarez R, Rubinos-Cuadrado G, Cabrera-Lacalzada C, Galindo-Morales R, Gullón-Blanco JA, González-Martín I (2009). Home mechanical ventilation: dependency and burden of care in the home. Arch Bronconeumol.

[17] Hwang MS, Lee MK, Song JR (2014). The factors affecting burdens and quality of life of the family caregivers of patients with rare and incurable diseases using home ventilators. Korean J Adult Nurs.

[18] Liu JF, Lu MC, Fang TP, Yu HR, Lin HL, Fang DL (2017). Burden on caregivers of ventilator-dependent patients: a cross-sectional study. Medicine.

[19] Jaracz K, Grabowska-Fudala B, Kleka P, Tomczak M, Smelkowska A, Pawlicka A, Górna K (2022). Development and Psychometric properties of the Caregiver Burden Scale in Polish caregivers of Stroke patients. Psychol Res Behav Manag.

[20] Evans R, Catapano MA, Brooks D, Goldstein RS, Avendano M (2012). Family caregiver perspectives on caring for ventilator-assisted individuals at home. Can Respir J.

[21] Kim CH, Kim MS (2014). Ventilator use, respiratory problems, and caregiver well-being in Korean patients with amyotrophic lateral sclerosis receiving home-based care. J Neurosci Nurs.

[22] Kaub-Wittemer D, Steinbüchel Nv, Wasner M, Laier-Groeneveld G, Borasio GD (2003). Quality of life and psychosocial issues in ventilated patients with amyotrophic lateral sclerosis and their caregivers. J Pain Symptom Manage.

[23] Vitacca M, Escarrabill J, Galavotti G, Vianello A, Prats E, Scala R, Peratoner A, Guffanti E, Maggi L, Barbano L, Balbi B (2007). Home mechanical ventilation patients: a retrospective survey to identify level of burden in real life. Monaldi Arch Chest Dis.

[24] Chiò A, Gauthier A, Calvo A, Ghiglione P, Mutani R (2005). Caregiver burden and patients’ perception of being a burden in ALS. Neurology.

[25] Siciliano M, Santangelo G, Trojsi F, Di Somma C, Patrone M, Femiano C, Monsurrò MR, Trojano L, Tedeschi G (2017). Coping strategies and psychological distress in caregivers of patients with amyotrophic lateral sclerosis (ALS). Amyotroph Lateral Scler Frontotemporal Degener.

[26] Marcus EL, Jacobs JM, Stessman J (2023). Prolonged mechanical ventilation and caregiver strain: home vs. long-term care facility. Palliat Support Care.

[27] Rossi Ferrario S, Zotti AM, Zaccaria S, Donner CF (2001). Caregiver strain associated with tracheostomy in chronic respiratory failure. Chest.

[28] Adelman RD, Tmanova LL, Delgado D, Dion S, Lachs MS (2014). Caregiver burden: a clinical review. JAMA.

[29] van Kesteren RG, Velthuis B, van Leyden LW (2001). Psychosocial problems arising from home ventilation. Am J Phys Med Rehabil.

[30] Yotani N, Ishiguro A, Sakai H, Ohfuji S, Fukushima W, Hirota Y (2014). Factor-associated caregiver burden in medically complex patients with special health-care needs. Pediatr Int.

[31] Boettcher J, Denecke J, Barkmann C, Wiegand-Grefe S (2020). Quality of Life and Mental Health in mothers and fathers caring for children and adolescents with rare diseases requiring long-term mechanical ventilation. Int J Environ Res Public Health.

[32] Del-Pino-Casado R, Frías-Osuna A, Palomino-Moral PA, Ruzafa-Martínez M, Ramos-Morcillo AJ (2018). Social support and subjective burden in caregivers of adults and older adults: a meta-analysis. PLoS ONE.

[33] Liang HY, Lee MD, Lin KC, Lin LH, Yu S (2022). Determinants of the health care service choices of long-term mechanical ventilation patients: applying andersen’s behavioral model. PLoS ONE.

[34] Nadig N, Huff NG, Cox CE, Ford DW (2016). Coping as a multifaceted construct: associations with psychological outcomes among family members of mechanical ventilation survivors. Crit Care Med.

[35] Kwiatosz-Muc M, Kopacz B, Fijałkowska-Nestorowicz A (2023). Quality of life and stress levels in patients under Home Mechanical Ventilation: what can we do to improve functioning patients at home? A Survey Study. Int J Environ Res Public Health.

[36] Pérez-Cruz M, Parra-Anguita L, López-Martínez C, Moreno-Cámara S, Del-Pino-Casado R (2019). Coping and anxiety in caregivers of dependent older adult relatives. Int J Environ Res Public Health.

[37] Cruzado JA, de la Elvira MJ (2013). Coping and distress in caregivers of patients with disorders of consciousness. Brain Inj.

[38] Kühnlein P, Kübler A, Raubold S, Worrell M, Kurt A, Gdynia HJ, Sperfeld AD, Ludolph AC (2008). Palliative care and circumstances of dying in German ALS patients using non-invasive ventilation. Amyotroph Lateral Scler.

[39] Tramonti F, Barsanti I, Bongioanni P, Bogliolo C, Rossi B (2014). A permanent emergency: a longitudinal study on families coping with amyotrophic lateral sclerosis. Fam Syst Health.

[40] Petrinec A (2017). Post-intensive Care Syndrome in Family decision makers of long-term Acute Care Hospital patients. Am J Crit Care.

